# Physiological and Therapeutic Properties of Mercury

**Published:** 1878-08

**Authors:** 


### Physiological and Therapeutic Properties of Mercury.

The following notice of Dr. Hallopeau's thesis appeared in *La France Medicale* of June 1, 1878:

The subject is an extensive one. To relate the very eventful history of mercury; to give the positive ideas in a chemical and physiological point of view, which are possessed at the present day relative to the absorption, circulation and elimination of this agent; to study its action on the organism by the aid of experimental and clinical research; and finally to point out and justify, in the best possible manner its therapeutic applications, has been the great aim and object of the author.

In the first place he shows by the aid of quotations from authors of very remote periods how mercury was first introduced in the materia medica, the oppositions to it, the difficulties it had to surmount, the prodigious abuse of it made by empirics, and even certain physicians. Thus, Torella, Vigo, Nicolas, Masses, Frascator, Ulrich de Hutten, Antonine Musa, Brassavola, Fallope, Paré and a good many others came respectively to overwhelm it with their condemnations or heap upon it their praises. At the expiration of the sixteenth century its triumph appeared to be assured, and indeed it reigned without opposition in the therapeutics of syphilis, until the end of the eighteenth century; but at this time it suddenly became the object of violent and multiplied attacks. It seemed to undergo the counter stroke of the revolutionary tempest, and was disputed like all the traditions bequeathed by the ancients. Anti-mercurial schools were seen to rise on all sides. Broussais discarded it like all other specifics; Auzais-Turenne opposed it with the object of substituting syphilization; Hermann, of Vienna, went farther and denied the existence of constitutional syphilis, ascribing to mercurialism all the symptoms of the disease. Curious to relate, this strange doctrine found ahderents in Germany. Other and more reasonable opposers contented themselves with saying that syphilis tends naturally to cure, that mercury does not modify its course or diminish its gravity, in consequence of which its administration is superfluous and dangerous. Notwithstanding all these attacks, the use of mercury in syphilis is to-day almost universally acknowledged. Thanks to our distinguished Ricord, it is employed by almost every physician in the land, and continues to render every day, the greatest service to humanity.—*Cin. Lancet and Clinic.*

